# Pulmonary injury following exposure to amorphous silicon dioxide nanoparticles in *Golden Syrian Hamsters*

**DOI:** 10.3389/ebm.2026.10793

**Published:** 2026-01-26

**Authors:** Rachel P. Renda, Joseph M. Cerreta

**Affiliations:** College of Pharmacy and Health Sciences, St. John’s University, Queens, NY, United States

**Keywords:** apoptosis, inhalation, nanoparticles, pulmonary, silicon dioxide

## Abstract

Amorphous silicon dioxide nanoparticles (SiO_2_ NPs) are abundant within the earth’s crust and can be released into the air through industrial and manufacturing activities. Such materials are often used in industrial processes, in pharmaceutical and in the cosmetic industries. Amorphous SiO_2_ NPs are pulmonary toxicants; however, the mechanism of toxicity is uncertain. In the current study, toxicity of SiO_2_ NPs was assessed using inhalation exposure in an *in vivo* system to study a possible mechanism of pulmonary injury. Golden Syrian Hamsters were divided into 4 groups: 1- room air control, 2- vehicle control, 3- low concentration (6 mg/m^3^) and 4- high concentration (12 mg/m^3^). Hamsters were treated for 4 h a day for 8 days. Bronchoalveolar Lavage Fluid (BALF) analysis found increases in total cell counts (p < 0.0001), neutrophils (p < 0.0001), lymphocytes (p < 0.001), eosinophils (p < 0.01), multinucleated macrophages (p < 0.01), total protein (p < 0.0001), alkaline phosphatase (p < 0.0001), and lactate dehydrogenase (p < 0.001) in the high concentration group. Histopathological analysis found an increase in air space, quantified by Mean Linear Intercept (p < 0.0001), and a significant increase in TUNEL positive cells (p < 0.001), in the high concentration group. SEM and TEM found structural alterations to the lung tissue including increase in the number holes in the alveolar walls and in apoptotic bodies within tissue. Caspase 3 (p < 0.05), and 8 (p < 0.05), were significantly increased along with cellular inflammation markers TNF-α (p < 0.05), and HSP70 (p < 0.05) in the high concentration group. Results of the study indicate exposure to SiO_2_ NPs may induce extrinsic apoptotic pathway, leading to tissue damage and significant airspace enlargement.

## Impact statement

Humans are exposed to amorphous SiO_2_ nanoparticles, occupationally, every day. There is limited research on the injury caused by inhalation of these materials and with their increasing use. With great focus on crystalline silica inducing silicosis there has been limited research on the amorphous form and its potential for toxicity. Often the amorphous form is deemed safe. This study provides an analysis of injury after exposure to amorphous silicon dioxide nanoparticles and evaluation of an *in vivo* cell death mechanism that may occur when exposed beyond OSHA’s limits.

## Introduction

Silica Nanoparticles (NPs) are composed of silicon dioxide (SiO_2_), one of the most abundant compound within the Earth’s crust [1, 2]. Silica exists naturally in a crystalline (quartz) form and in an amorphous form (diatomaceous earth) while amorphous silica can also be synthetically produced in nano-sized forms [3]. Silica nanoparticles are particularly desirable for use in consumer products, as industrial additives, and in drug delivery because they remain stable over a wide range of temperatures, in organic solvents, and under acidic conditions [4].

Silica exposure can be harmful for those in an occupational setting, as silicosis is a known occupational disease linked to inhalation of crystalline SiO_2_ NPs [5]. While crystalline silica is widely recognized as the more toxic form of inhaled silica, exploring the effects of exposure to amorphous SiO_2_ NPs remains essential, as they are widely used and pose risks to those in an occupational setting [5].

SiO_2_ NPs and various other oxide NPs have been shown to cause pulmonary injury [6–8]. Exposure to crystalline SiO_2_ NPs has shown lysosomal disruption, blockage of autophagic flux, ROS generation, and apoptosis [7, 8]. Mice exposed to amorphous SiO_2_ NPs through intratracheal instillation were found to have increased markers of pulmonary inflammation, increased neutrophils, and increased macrophage production of inflammatory mediators [6]. The current guidelines set by The Occupational Safety and Health Administration’s (OSHA) Permissible Exposure Limit (PEL) for exposure to amorphous SiO_2_ NPs in an 8-h period is 6 mg/m^3^.

Nanoparticles can enter the body through several routes including, ingestion, dermal exposure with the most common pathway being inhalation. To understand the effects in the lungs after exposure to amorphous SiO_2_ NPs this study design exposed animals through a full body exposure chamber, as it is most representative of a real-time exposure. Though many inhalation studies expose animals through intratracheal instillation there are advantages to the whole-body exposure method. The whole-body exposure chamber provides a pulmonary response to a typical exposure route with no discomfort to the animal [9–11]. Within the whole-body exposure chamber animals may receive an incidental exposure of NPs via the oral route due to grooming. Kwon et al., found intake of orally ingested SiO2 NPs are excreted in their intact particle form via feces [12].

The animal model used in the current study was the Golden Syrian Hamster that have been used to study models of pulmonary fibrosis, chronic bronchitis, emphysema and SARS-CoV-2 [13–19].

Apoptosis is a cell death mechanism that has been seen with exposure to crystalline silica [8]. Apoptosis can be broken into two pathways, an intrinsic mitochondrial-dependent, pathway and an extrinsic death-receptor based pathway [20]. The mitochondrial dependent intrinsic pathway is triggered by growth factor deprivation and cellular stress. Downstream events include the release of Cytochrome C from the mitochondria, binding to APAF-1 to form the apoptosome, activation of caspase 9 and cleavage of caspase 3 [21]. The alternate or extrinsic pathway is triggered by extracellular signals that death receptors on the plasma membrane recognize [21]. Death receptors then recruit adaptor molecules (FAS-associated death domain and TNFRS1A-associated death domain) [21]. The adaptors activate pro-caspase 8 to form, caspase 8 that then interacts to cleave caspase 3. Caspase 3 executes cell death through apoptosis [21].

The aim of this study is to explore the resulting injury after exposure to amorphous SiO_2_ NPs in Golden Syrian Hamsters. This study hypothesizes that inhaled amorphous SiO_2_ at the level of OSHA’s PEL (6 mg/m^3^) will show minimal pulmonary injury, but inhalation of the higher concentration (12 mg/m^3^) will show internalization of amorphous SiO_2_ NPs, activation of apoptosis and pulmonary injury to respiratory epithelium.

## Materials and methods

### Materials

Silicon dioxide nanoparticles were purchased from Nanostructured and Amorphous Materials, Inc. (Katy, TX). Methyl Green and Vectabond® Reagent were acquired from Vector Laboratories, Inc. (Burlingame, CA). Albumin Standard (2 mg/mL), Nanosphere™ Size Standards, Pierce™ BCA Protein Assay Kit, Pierce Protease and Phosphatase Inhibitor Mini Tablets, PROTOCOL® Hema 3® Stain Set, and Zeta Potential Standard were obtained from Thermo Fisher Scientific (Waltham, MA). ApopTag® Plus Peroxidase *In Situ* Apoptosis Detection Kit and Proteinase K were obtained from EMD Millipore Corp. (Burlington, MA). Caspase 3, Caspase 8, and Alkaline Phosphatase colorimetric assay kits were obtained from Abcam (Waltham, MA). LDH Cytotoxicity Assay Kit was obtained from Cayman Chemicals Inc., (Cambridge, MA, United States). Lamelli Sample Buffer, Mini-PROTEAN TGX Gels (7.5%, 12%, 4–20% and Any KD™), nonfat dry milk and Precision Plus Protein Dual Color Standards were purchased from Bio-Rad Laboratories, Inc. (Hercules, CA). MagicMark XP Western Protein Standard was purchased from Invitrogen, Thermo Fisher Scientific (Carlsbad, CA). Amersham ECL Prime Western Blotting Detection Reagents were purchased from Global Life Science Solutions Operations UK Ltd., Cytiva (Buckinghamshire, UK). Antibodies to β-Actin, Caspase 3, 8, 9, and HSP70 were purchased from Cell Signaling Technology, Inc. (Danvers, MA). TNF-α ELISA and Cytochrome C ELISA KIT Abcam (Waltham, MA). Glutaraldehyde was purchased from Electron Microscopy Sciences (Hatfield, PA). Lead nitrate, osmium tetroxide, sodium cacodylate, sodium citrate, and uranyl magnesium acetate were purchased from Ted Pella, Inc. (Redding, CA).

### Methods

#### Nanoparticle characterization

The average size of SiO_2_ NPs according to the manufacturer was 20 nm with a purity of 99%. SiO_2_ NPs were directly measured using TEM, by loading particles on a formvar coated copper grid. Nanosphere™ Size Standards with a mean diameter of 100 nm ± 4 nm were used as a calibration standard. TEM micrographs were analyzed using ImageJ software. The mean hydrodynamic diameter of SiO_2_ NPs in water was determined by dynamic light scattering (DLS using a Delso Nano C Particle Size Analyzer (Beckman Coulter Inc.). The zeta potential of SiO_2_ NPs was measured using the Malvern Zetasizer Nano ZS.

#### Animals

Female Golden Syrian Hamsters, age 7–8 weeks were obtained from Envigo (Indianapolis, IN). Animals were housed at St. John’s Universities Animal Care Center, Queens, NY, an AAALAC approved facility. Upon arrival animals were given a seven-day acclimation period. Hamsters were kept under a controlled environment with a 12-h light-dark cycle and provided a standard laboratory diet and water *ad libitum*. Animal studies were carried out under an Institutional Animal Care and Use Committee (IACUC) approved protocol. 9 animals per treatment group were used.

#### Exposure system and experimental design

Studies were carried out using 4 animal groups: room air control (filtered room air), vehicle control (sterile aerosolized water), low concentration SiO_2_ NPs (6 mg/m^3^) and high concentration SiO_2_ NPs (12 mg/m^3^). Animals were exposed for 4 h a day for 8 days in a whole-body exposure chamber. In this repeated exposure model, animals were exposed to a concentration of either 6 mg/m^3^ or 12 mg/m^3^ amorphous SiO_2_ NPs, continuously over the 4-h exposure period. The concentration of 6 mg/m^3^ was chosen as it represents the PEL (OSHA) and TWA (NIOSH), 12 mg/m^3^ was chosen as it is double the regulatory level. The high concentration (12 mg/m^3^) was selected to represent approximately twice the current occupational exposure limit for amorphous SiO_2_ NPs. This approach intended to examine pulmonary responses that could arise under conditions exceeding regulatory standards, during accidental release or short term over exposures that may occur in occupational settings. This additional exposure concentration provides a dose response relationship relative to the established exposure limit. Animals were euthanized with an overdose of ketamine (800 mg/kg) and xylazine (40 mg/kg) via an intraperitoneal route 24 h after the last exposure.

To form an aerosol, an 8-jet nebulizer (CH Technologies, Inc., NJ) attached to a collision jar was used with air supplied from a compressed air tank. SiO_2_ Nanoparticle suspensions were prepared in advance using sterile distilled water and sonicated prior to treatment. To measure the concentration of particles in the chamber air samples were taken every minute and measured for mass aerosol concentration (mg/m^3^) using a NanoScan SMPS 3910 (TSI, Inc., MN).

#### Analysis of bronchoalveolar lavage fluid (BALF)

Post euthanasia, lungs were lavaged with cold Dulbecco’s phosphate buffer saline and the bronchoalveolar lavage fluid (BALF) was collected. BALF was centrifuged and the acellular component was aliquoted out and stored at −80 °C for further use. The cellular component was resuspended with RPMI media and used to determine differential cell counts of leukocytes. Smears of the BALF were prepared using cytocentrifugation and samples spun, dried and stained with PROTOCOL HEMA 3. Cells were counted and categorized as: macrophages, multinucleated macrophages, neutrophils, lymphocytes, and eosinophils. Cell counts were carried out as previously described [22].

The acellular component of the BALF was analyzed for total protein as measured using the Pierce BCA Protein Assay Kit, LDH and ALP levels using the LDH Colorimetric Assay Kit and Alkaline Phosphatase Colorimetric Kit (Abcam). LDH and ALP release was calculated from the generated standard curves.

#### Light microscopy

For morphologic examination, following euthanization, lungs were inflated by insufflation with 10% Neutral Buffered Formalin. Fixed tissue was dehydrated, cleared, and embedded in Paraplast X-TRA. Paraffin tissue blocks were sectioned using a microtome. Sections were cut, ∼5 microns thick, placed on glass slides, deparaffinized in xylene and rehydrated in increasing concentrations of ethanol to distilled water. Tissue sections were stained with hematoxylin and eosin, dehydrated in ethanol and cleared with xylene and mounted with cover glass using permount.

#### Mean linear intercept

Photomicrographs taken of H&E-stained sections were analyzed for differences in alveolar size by calculating the Mean Linear Intercepts. Mean Linear Intercept was measured by the line intersection method and calculated from 20 random fields of view per animal [23]. Micrographs were analyzed using ImageJ software.

#### TUNEL assay

A TUNEL Assay was carried out using the ApopTag® Plus Peroxidase *In Situ* Apoptosis Detection Kit. Slides were prepared as stated above, deparaffinized, and rehydrated. Tissue sections were pretreated with Proteinase K and washed in distilled water. Slides were then quenched in 3% H_2_O_2_ and washed in PBS. The TUNEL Assay protocol (ApopTag®) was followed as directed by the manufacturer. Photomicrographs of tissue sections were taken, and apoptotic bodies were counted from 20 random fields of view.

#### Electron microscopy

Alterations to the ultrastructural morphology of the cells and tissue of the lung was evaluated following exposure to silicon dioxide NPs using scanning electron microscopy (SEM) and transmission electron microscopy (TEM). After animals were euthanized, lungs were fixed by insufflation with 3% glutaraldehyde. For SEM, samples following fixation were washed, dehydrated with increasing concentrations of acetone, and dried using the critical point method. Samples were mounted on aluminum stubs, and sputter coated with platinum and palladium. The samples were examined using a scanning electron microscope. For TEM, lung tissue blocks were cut, post-fixed in osmium tetroxide, stained in uranyl acetate, dehydrated in acetone and embedded in LX112-Ardalite resin mixture. Following polymerization, tissue blocks were sectioned and stained with uranyl acetate and lead citrate. Samples were examined using a transmission electron microscope.

#### Immunoblot analysis

Western blot analysis of Caspase 9, Caspase 8, Caspase 3, and HSP70 were carried out using the Bio-Rad protein guide protocol (Bio-Rad) and the Abcam western blot protocol (Abcam). Lung tissue was collected, washed in DPBS, frozen and stored in liquid nitrogen until use. Lung tissue was homogenized in cold RIPA buffer containing a protease and phosphatase inhibitor cocktail. Total Protein was determined using Pierce BCA Protein Assay Kit. Proteins were denatured and then separated using electrophoresis. Resolved proteins were transferred to a PVDF membrane, blocked in 5% (w/v) non-fat dry milk in Tris Buffered Saline (TBST) for 1 h at room temperature or overnight at 4 °C. TBST washed membranes were incubated with primary antibody for 3 h at room temperature or overnight at 4 °C. All primary antibodies were monoclonal derived from rabbit and an anti-rabbit IgG HRP-linked secondary antibody (1:2000) was used to probe the membranes. The following conditions were used (% SDS PAGE gel, amount of total protein, primary antibody incubation time, blocking time, and antibody dilutions), respectively for Caspase 9 (4–20%, 90 μg, 3 h, overnight, 1:1000), for Caspase 8 (4–20%, 90 μg, overnight, 1 h, 1:1000), for Caspase 3 (any KD™, 90 μg, overnight, 1 h, 1:1000), and for HSP70 (4–20%, 90 μg, 3 h, overnight, 1:1000). β-Actin, used as a loading control, was measured by probing with Anti-IgG B actin (1:5000) and incubated for 1 h. After incubation with the secondary antibody (anti-rabbit IgG HRP-linked), chemiluminescence was detected using Amersham™ ECL™ Prime Western Blotting Detection Reagent and membranes were photographed using a digital imager. Images were analyzed by densitometry using ImageJ software.

#### ELISA

TNF-α and Cytochrome C levels were determined in lung samples from control and treated animals using a sandwich ELISA technique kit (TNF-α Rat SimpleStep ELISA® Kit or Mouse/Rat Cytochrome C SimpleStep ELISA® Kit, Abcam). Concentration of lung homogenate used was based on manufacturer protocols and provided protocols were followed. Protein levels were calculated from the generated standard curves.

#### Statistical analysis

Data were presented as the mean ± SEM. Statistical analyses were carried out using Graph Pad Prism 9 with significance tested among and between groups using one-way analysis of variance (ANOVA) followed by Tukey’s Post Hoc analysis. Differences between treated groups were significant at the 95% level (P < 0.05) and noted based on the following *P < 0.05, **P < 0.01, ***P < 0.001, ****P < 0.0001. Room air controls did not statistically differ from the vehicle controls; therefore, only vehicle control data are presented in the main figures, with room air control data provided in the Supplementary Material.

## Results

### Nanoparticle characterization

**FIGURE 1:**
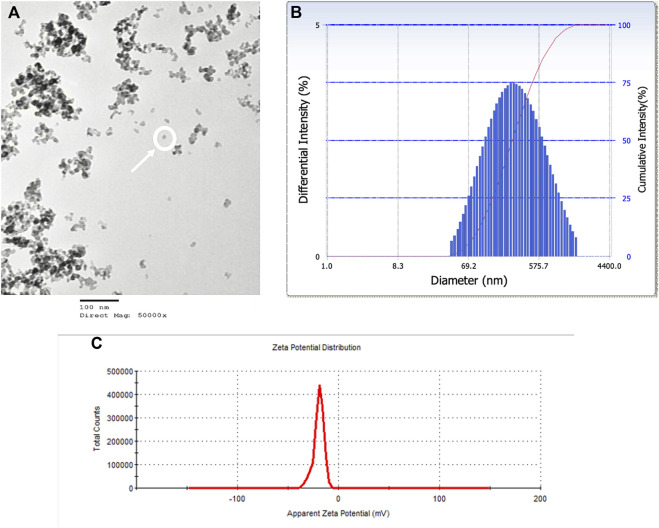
SiO_2_ NPs were characterized by TEM, DLS, and Zeta Potential. **(A)** A TEM micrograph of SiO_2_ NPs suspension on a copper coated grid. The micrograph shows single particles (encircled) among particle clumps. Average particle size measured 20 nm. **(B)** Representative distribution of particle size in aqueous suspension of amorphous SiO_2_ NPs measured using DLS. The average hydrodynamic diameter was measured as 226 nm. **(C)** Zeta Potential distribution of amorphous SiO_2_ NPs in water was measured as −20 mV.

SiO_2_ NPs were characterizing using TEM to determine particle size (Figure 1A), DLS to determine mean hydrodynamic diameter (Figure 1B) and Zeta Potential was measured (Figure 1C). The average individual particle size was measured to be 20 nm and had a spherical shape. The mean hydrodynamic diameter was 226 nm in water. The Zeta Potential of SiO_2_ NPs in water was −20 mV.

#### BALF analyses

**FIGURE 2:**
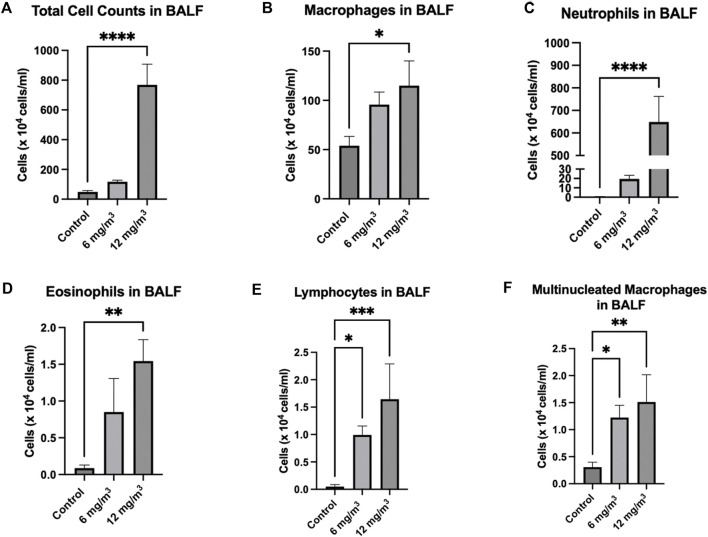
Total Cells and leukocytes in the bronchoalveolar lavage fluid (BALF) of Golden Syrian Hamsters were counted to determine if inflammation was occurring after exposure to inhaled SiO_2_ NPs. The cells were re-suspended in 1 mL of RPMI media followed by determination of total cell numbers **(A)**. Total Cell numbers **(A)** were significantly increased by 16-fold in the 12 mg/m^3^ treated group. Differential cell count **(B–F)** slide smears were prepared from the BALF of cells from the lung exposed to 6 and 12 mg/m^3^ SiO_2_ NPs for 4h/day for 8 days. Macrophages **(B)** of treated groups were not changed when compared to control. Neutrophils **(C)**, Eosinophils **(D)**, Lymphocytes **(E)**, and Multinucleated Macrophages **(F)** were significantly increased in the 12 mg/m^3^ treated group when compared to control. N = 5.

Exposure to SiO_2_ NPs resulted in significant increase in total number of cells in the BALF. Cell numbers were increased from 67.9 × 10^4^ cells/mL in the controls to 119.6 × 10^4^ cells/mL and 770.1 × 10^4^ cells/mL in the treated groups (6 mg/m^3^ and 12 mg/m^3^ respectively) (Figure 2A; Supplementary Figure S1). Inhalation of SiO_2_ NPs did not cause a significant change in macrophages (Figure 2B; Supplementary Figure S1) but led to a significant increase in neutrophils from 4.3 × 10^4^ cells/mL in controls to 19.4 × 10^4^ cells/mL in 6 mg/m^3^ and 648.9 × 10^4^ cells/mL in 12 mg/m^3^ (Figure 2C; Supplementary Figure S2). Lymphocyte numbers were also significantly increased in the BALF of treated animals as compared to controls. Eosinophils were increased only in the 12 mg/m^3^ group by 7-fold (Figure 2D; Supplementary Figure S1). The numbers of lymphocytes in the BALF of treated animals were 20-fold higher in 6 mg/m^3^ and 33-fold higher in 12 mg/m^3^ (Figure 2E; Supplementary Figure S1). Multi nucleated macrophages were significantly increased by 4-fold in 6 mg/m^3^ and 5-fold in 12 mg/m^3^ (Figure 2F; Supplementary Figure S1).

**FIGURE 3:**
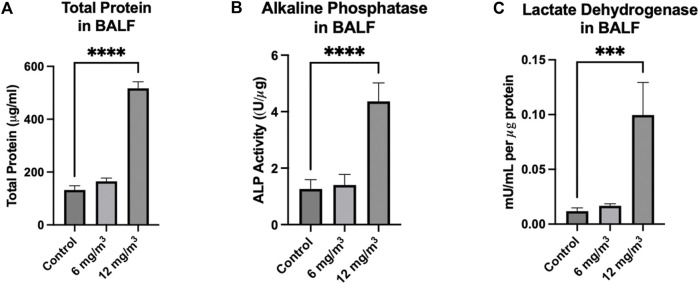
Markers measured in the BALF of Golden Syrian Hamsters from lungs exposed to 6 and 12 mg/m^3^ SiO_2_ NPs for 4h/day for 8 days were Total Protein (TP) Alkaline Phosphatase (ALP), and Lactate Dehydrogenase (LDH). **(A)** TP, **(B)** ALP), and **(C)** LDH were all significantly increased in the 12 mg/m^3^ treated group when compared to vehicle control by 4-fold, 9-fold and 3-fold, respectively. N = 4.

Total Protein in the BALF was significantly increased from 133 ± 15 μg/mL in the controls to 517 ± 25 μg/mL in the 12 mg/m^3^ group (Figure 3A; Supplementary Figure S3). ALP activity in the BALF was significantly elevated in the 12 mg/m^3^ group (4.4 ± 0.6 U/μg) when compared to vehicle control (1.3 ± 0.3 U/μg) (Figure 3B; Supplementary Figure S3). LDH activity in the BALF was significantly increased in the 12 mg/m^3^ group (99 × 10^−3^ ± 29 × 10^−3^ mU/mL), when compared to vehicle control (11 × 10^−3^ ± 2.9 × 10^−3^ mU/mL) (Figure 3C; Supplementary Figure S3).

### Morphological analysis using light and electron microscopy

#### Light microscopy and MLI

**FIGURE 4:**
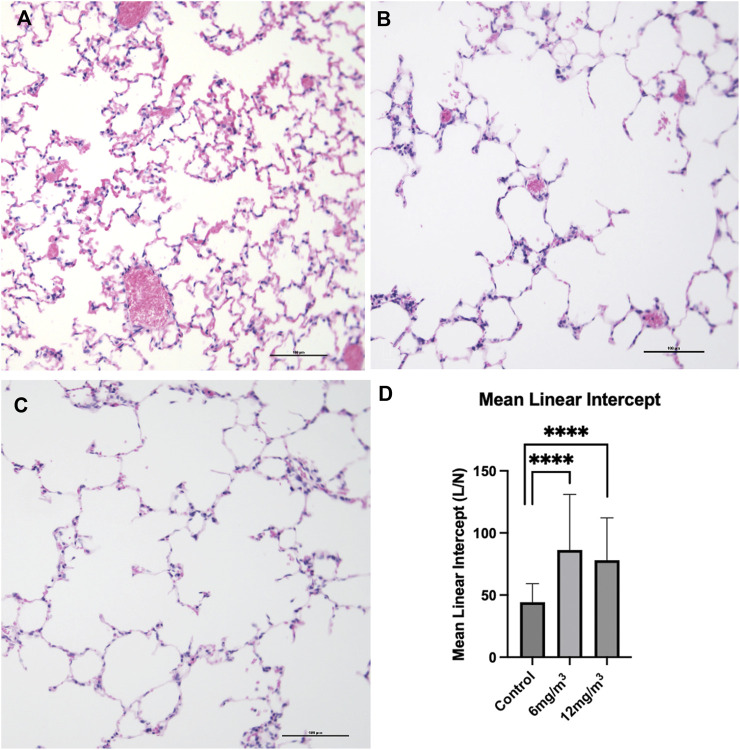
The photomicrographs are of formalin fixed, H&E-stained tissue sections from the lungs of **(A)** vehicle control (water), **(B)** 6 mg/m^3^ and **(C)** 12 mg/m^3^ SiO_2_ NP treated groups. The micrographs show progressive increase in air space quantified by Mean Linear Intercept **(D)**. Mean Linear Intercept was measured by the line intersection method and calculated from 20 random fields of view per animal. The Mean Linear Intercept of both 6 mg/m^3^ and 12 mg/m^3^ SiO_2_ NP treated animals was significantly increased when compared to vehicle control (water).

Lung tissue sections from vehicle control stained with H&E showed the architecture of parenchyma as seen in (Figure 4A; Supplementary Figure S4). Histopathological examination of lung tissue sections exposed to 6 mg/m^3^ or 12 mg/m^3^ SiO_2_ NPs showed increase in average airway space (Figures 4B,C). Air space size seen in all groups was quantified by Mean Linear Intercept. Tissue sections of both low and high concentration treated groups showed a significant increase in the Mean Linear Intercept from ∼44 μm in controls to ∼86 μm in the 6 mg/m^3^ treated group and to ∼78 μm in the 12 mg/m^3^ treated group (Figure 4D).

#### TUNEL Assay

**FIGURE 5:**
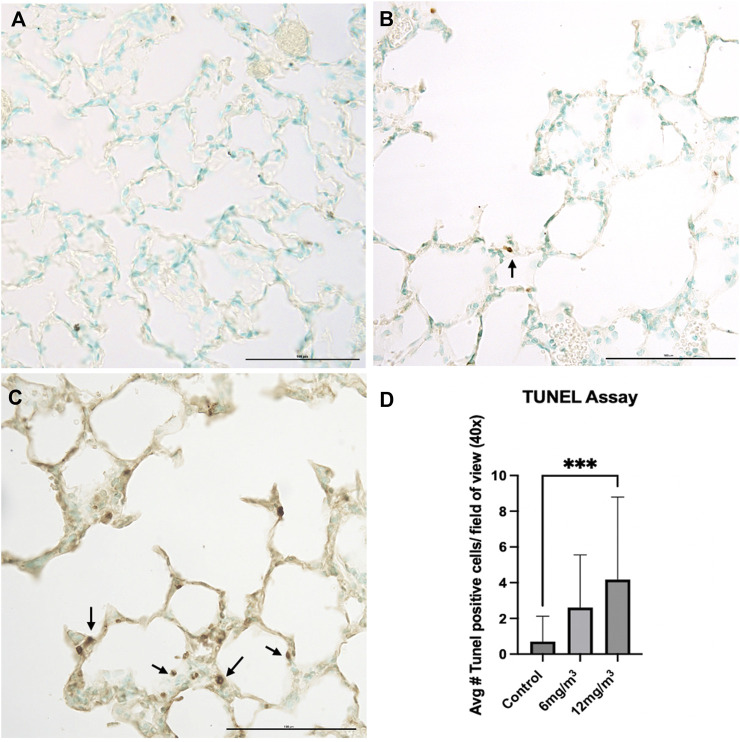
The photomicrographs are of formalin fixed tissue sections from the lungs of **(A)** vehicle control (water), **(B)** 6 mg/m^3^ and **(C)** 12 mg/m^3^ SiO_2_ NP treated groups. The TUNEL Assay-stained tissue sections for TUNEL positive cells (apoptotic bodies). The micrographs show an increase with increased concentration of NPs with a concomitant increase in the number of apoptotic bodies. Arrows indicate apoptotic bodies. **(D)** A histogram of the count from the TUNEL assays shows a significant increase in TUNEL positive cells in the high concentration SiO_2_ NP treated animals compared to vehicle control (water). The average TUNEL positive cells was based on calculated results of 20 random fields of view.

Histopathological examination of lung tissue sections exposed to 6 mg/m^3^ and 12 mg/m^3^ SiO_2_ NPs showed increase in numbers of apoptotic bodies when compared to control (Figures 5A–C; Supplementary Figure S5). Average TUNEL positive cells in the vehicle control group were less than 1, while average TUNEL positive cells in the 6 mg/m^3^ group had a mean of 3 TUNEL positive cells and the 12 mg/m^3^ group was significantly increased with a mean of 4 TUNEL positive cells (Figure 5D).

#### Electron microscopy

**FIGURE 6:**
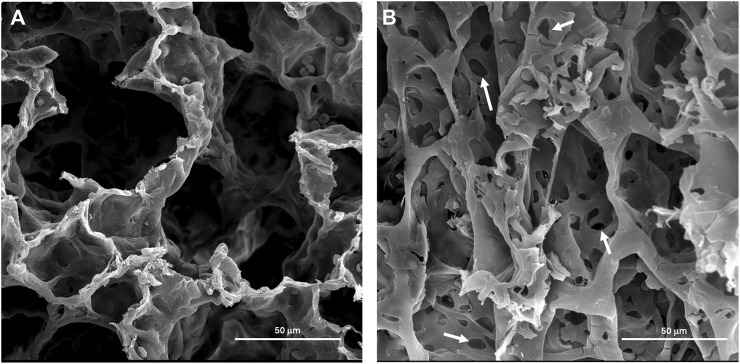
Representative scanning electron micrographs (SEM) of lung tissue from control and treated animals fixed in 3% glutaraldehyde and processed as described in methods. **(A)** The photomicrograph is from the lungs of a vehicle control (water) animal that shows typical architecture of the alveoli. **(B)** The lung tissue of an animal treated with 12 mg/m^3^ shows an atypical architecture of the alveoli with increased holes within the alveoli wall marked by arrows.

Scanning Electron Microscopy was carried out to determine surface morphological alterations after exposure to both 6 mg/m^3^ and 12 mg/m^3^ SiO_2_ NPs. Alveoli of vehicle control exposed animals had no pathological changes (Figure 6A). The 12 mg/m^3^ treated group had a distinct change in morphology, with an increase in holes within the alveolar walls and noticeably thin septa between the alveoli (Figure 6B).

**FIGURE 7:**
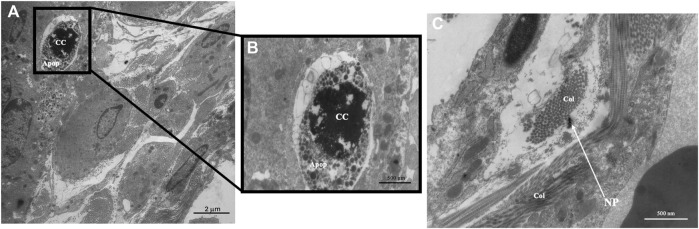
Representative transmission electron micrographs (TEM) of lung tissue from control and treated animals fixed in 3% glutaraldehyde and processed as described in methods. TEM photomicrographs of an interstitial region from a 12 mg/m^3^ treated animal showing **(A)** apoptotic bodies and the presence of collagen, original magnification ×5,000. **(B)** An enlargement of the region of apoptotic bodies, original magnification ×20,000. **(C)** An interstitial region showing the presence of collagen and SiO_2_ NPs, original magnification ×20,000. Key: Apoptotic Bodies (Apop), Chromatin Condensation (CC), Collagen (Col), Nanoparticles (NP).

Transmission Electron Microscopy was done to determine ultrastructural cellular alterations after exposure to SiO_2_ NPs. Within the interstitial region of the 12 mg/m^3^ treated animal, apoptotic bodies, chromatin condensation (Figures 7A,B) and possible SiO_2_ NPs (Figure 7C) were present. Control and treated animal tissue sections show intact Type II pneumocytes with lamellar bodies and mitochondria (Supplementary Figure S6).

#### Protein analysis of apoptosis and related cell markers

**FIGURE 8:**
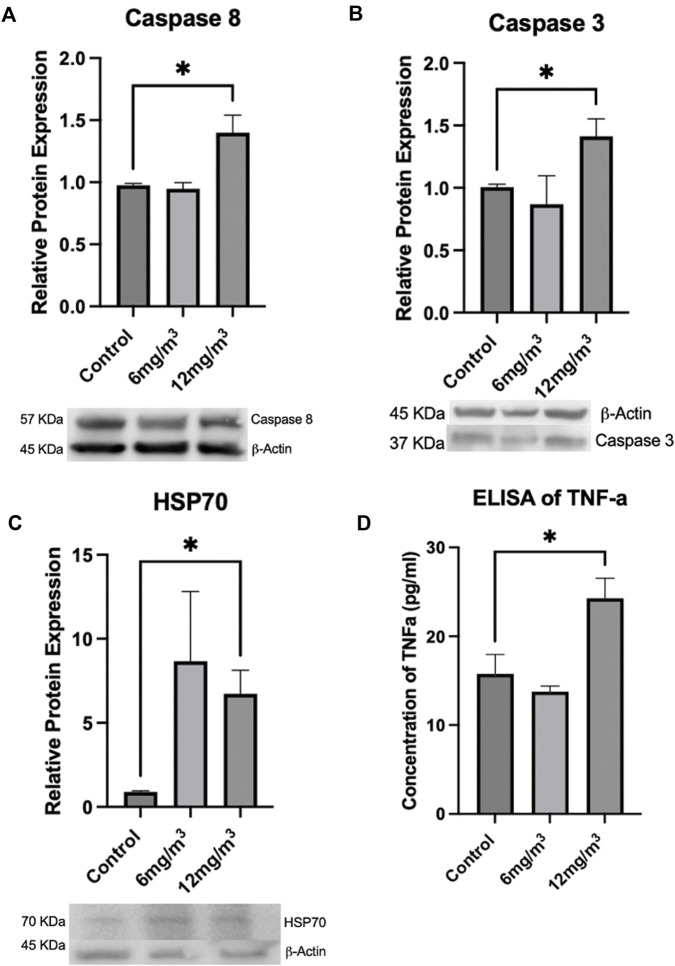
To determine if apoptosis was occurring, Caspases 8, 3, HSP70 and TNF-α were measured via Western Blot or ELISA. **(A)** Caspase 8 and **(B)** Caspase 3 were significantly increased in the 12 mg/m^3^ treated group when compared to vehicle control (water). **(C)** HSP70, a marker of cellular stress, was measured by Western Blot and was significantly increased in the high concentration group when compared to vehicle control (water). **(D)** An ELISA of TNF-α levels was significantly increased in the high concentration treated group when compared to vehicle control (water). For Western Blots and ELISAs n = 4.

Caspase 9 and Cytochrome C levels, both part of the intrinsic apoptotic pathway, were unchanged in either treatment group when compared to control (Supplementary Figure S7). Caspase 8, part of the extrinsic pathway of apoptosis, was not changed in the 6 mg/m^3^ group, but was significantly increased by 1.4-fold in the 12 mg/m^3^ treatment group (Figure 8A; Supplementary Figure S8). Caspase 3 protein expression was not changed in the 6 mg/m^3^ group but was significantly increased by 1.4-fold in the 12 mg/m^3^ treatment group (Figure 8B; Supplementary Figure S8). Inflammatory and cellular stress proteins TNF-α and HSP70 were measured. HSP70 levels were not changed in the 6 mg/m^3^ group but were significantly increased in the 12 mg/m^3^ group by 7.5-fold (Figure 8C; Supplementary Figure S8). TNF-α levels were not changed in the 6 mg/m^3^ group but were significantly increased in the 12 mg/m^3^ group by 1.5-fold (Figure 8D; Supplementary Figure S8).

## Discussion

Physical Characterization of a nanoparticle is important as physiochemical factors can alter their toxicity profile and action within a suspension, and the size and shape of a particle can influence its deposition in the lung [24]. To characterize the particle, the SiO_2_ NPs were evaluated for size, aggregation, and zeta potential using transmission electron microscopy (TEM) and dynamic light scattering (DLS). Both wet and dry states of the particle size were evaluated to consider particle-particle and particle-fluid interactions alone and in suspension [25]. SiO_2_ NPs measured by TEM were 20 nm while SiO_2_ NPs in water had a mean hydrodynamic diameter of 226 nm. The apparent increase in size in water may be due to possible agglomeration within the chamber that is a characteristic likely based on the reported hydrodynamic diameter and zeta potential of the nanoparticles [16]. Agglomeration of particles within a whole-body chamber has been seen in Tungsten NPs when deposited on grids and analyzed by TEM [16]. The zeta potential of SiO_2_ NPs was −20 mV in water, indicating stability with an anionic charge. Particles with a higher zeta potential have stronger electro-repulsion that can stabilize them preventing aggregation [26]. Negatively charged particles can easily move across mucus but have a difficult time crossing the cell membrane, often binding to a cell with positively charged surface proteins [26]. Such properties may inhibit NP cell entry through the cell membrane but may enhance numbers of inflammatory cells in the BALF.

Amorphous SiO_2_ NPs were used to induce injury and identify a possible cell death pathway. To mimic real life exposure to SiO_2_ NPs a whole-body exposure system was used. The selection of 4 h of exposure to SiO_2_ NPs per day was based on the OECD Guidelines for acute inhalation toxicity studies [27]. To study the mechanism of toxicity animals were exposed continuously for 8 consecutive days for 4 h per day to either 6 mg/m^3^ or 12 mg/m3 amorphous SiO_2_ NPs, that is consistent with other studies [16, 19]. The concentration of 6 mg/m^3^ was chosen based on OSHA’s Permissible Exposure Limit (PEL) and NIOSH’s Time Weighted Average (TWA) for amorphous silicon dioxide. The concentration of 12 mg/m^3^ was chosen as it is double the set regulatory level, to simulate over exposure events. This additional concentration provides a dose response relationship that helps define a margin of safety relative to the exposure limit. These concentrations may not only capture regulatory relevant, but also high-end exposure scenarios to create a more comprehensive risk characterization.

Following exposure to 12 mg/m^3^ amorphous SiO_2_ NPs total leukocyte counts in the BALF were significantly increased indicating an inflammatory response [28]. The animal group exposed to 12 mg/m^3^ had significant increases in total cell counts, neutrophils, lymphocytes, eosinophils, and multinucleated macrophages. A significant increase in cell types other than macrophages indicates an acute inflammatory response at 24 h after the last exposure [28]. Increased neutrophils may have the ability to shift protease/antiprotease balance in the lung leading to tissue injury [29]. Total Protein (TP), Lactate Dehydrogenase (LDH) and Alkaline Phosphatase (ALP) were all significantly increased in the BALF of the group treated with 12 mg/m^3^ SiO_2_ NPs. Total protein elevations can be attributed to injury of the alveolar-capillary barrier [30]. Increased LDH, a cytoplasmic enzyme, confirms the loss of cellular membrane integrity with an increased ALP, being a measure of damage to the alveolar epithelium [28, 31].

To date, the literature does not report any histologic changes comparable to those observed in this study. Lungs exposed to crystalline silica NPs have shown airway inflammation, collagen deposition and fibrosis [32, 33]. Histopathological examination of pulmonary tissue sections in the current study shows damaged alveolar walls with increased airspace size, quantified by Mean Linear Intercept. The observed increase in airspace size is attributed to cell death and subsequent alveolar wall destruction, resembling histologic features of emphysemic lungs [34]. Lung tissue sections stained using the TUNEL method have a significant increase in TUNEL positive cells present in the 12 mg/m^3^ treated animal group. Kong et al, (2020) found increase TUNEL positive cells alongside caspase activation to confirm apoptosis occurring in mice [35].

Destruction of the alveolar septal tissue is a characteristic of emphysema [36]. Scanning Electron Microscopy (SEM) micrographs of the 12 mg/m^3^ group show thinning of the interalveolar septa, with increase in holes within the alveolar walls [37]. Lung sections from TEM images of the 12 mg/m^3^ treated animals showed, apoptotic bodies, damaged interstitial cells, and SiO_2_ NPs. Mitochondria appear unchanged showing tightly packed cristae with parallel alignment, higher matrix density, and visible matrix dense granules as opposed to injured mitochondria that would show increases in intermembrane spaces and shape alterations [38]. No change to Type I or II pneumocytes was found in the 12 mg/m^3^ treated animals. Kantari and Walczak [39] found that the process of caspase 3 cleavage by caspase 8 can be blocked by XIAP (X-linked inhibitor of apoptosis) in Type II pneumocytes, that suggests while apoptosis is occurring in other cells, it may not be occurring in Type II pneumocytes [39]. This may explain lack of pathology in Type II pneumocytes in the 12 mg/m^3^ TEM micrographs. Interstitial regions within the 12 mg/m^3^ treated animals had visible apoptotic bodies with SiO_2_ NPs present.

To confirm that the mechanism of apoptosis was activated, Cytochrome C, Caspases 9, 8 and 3 were measured. Cytochrome C and Caspase 9 levels did not change, while Caspases 3, and 8 were significantly increased in the 12 mg/m^3^ group when compared to controls. Based on the literature crystalline silica has been found to increase Caspase 3, 9 and Cytochrome C release that supports an intrinsic pathway [40]. Results from the current study suggest an extrinsic pathway of apoptosis is the primary mechanism for cell death occurring after exposure to amorphous SiO_2_ NPs. The extrinsic pathway is typically initiated by activation of cell surface receptors. Macrophages and neutrophils present after exposure to SiO_2_ NPs will phagocytize the NPs leading to interactions that produce inflammatory factors such as TNF-α [41]. When TNF-α is released, it can bind to a transmembrane receptor to initiate apoptosis leading to Caspase 8 and eventual Caspase 3 cleavage [42].

The lack of change in Caspase 9 and Cytochrome C may be attributed to an increase in HSP70, that can block mitochondrial translocation, activation of Bax, and prevent mitochondrial membrane permeabilization, Cytochrome C release, and Caspase 9 cleavage [43, 44]. Results from the current study including increased TNF-α, Caspase 3, 8, HSP70 along with no change in Caspase 9 or Cytochrome C levels support both the activation of the extrinsic pathway while eliminating the intrinsic pathway as the mechanism of apoptosis [43, 44].

The data from this study suggests that after exposure to amorphous SiO_2_ NPs inflammatory cells such as macrophages, neutrophils, eosinophils, and lymphocytes are recruited. As these inflammatory cells are activated, they begin to release cytokines and proteases [45]. Elastic fibers, that act to maintain alveolar wall integrity, require balance between elastase and anti-elastase, while an imbalance can lead to tissue damage and resulting air space enlargement [45]. Once activated, neutrophils can release neutrophil elastase (NE), that is normally neutralized by alpha-1-antiprotease (A1AP) [45]. When inflammation is present the balance between NE and A1AP can be altered leading to destruction of lung tissue by unopposed NE breaking down elastin in the lung septa [45]. It may be possible that resulting inflammation from exposure to amorphous SiO_2_ NPs triggers such an imbalance in protease-antiprotease balance leading to resulting TUNEL positive cells, elevated apoptotic markers, increased airspace (reported by increased MLI) and increased hole size and number within the alveolar walls, along with thinning of the alveolar septa seen in SEM. Damage to lung tissue found in this study is comparable to emphysemic lung architecture, that is reported as abnormal permanent dilation of airspaces [45].

**FIGURE 9:**
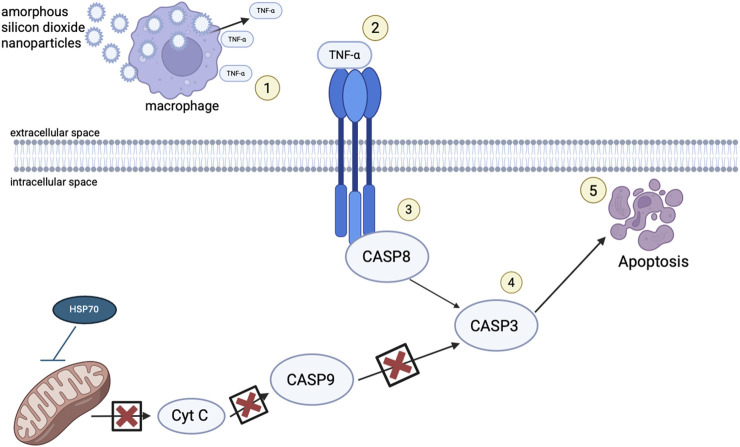
Schematic representation of the proposed mechanism of injury following exposure to SiO_2_ NPs. (1) After exposure to SiO_2_ NPs through inhalation, the NPs flow through the respiratory tract until they reach the alveoli where they are met by macrophages and neutrophils and they are phagocytized. During particle overload, and lack of mucociliary clearance, macrophages signal an inflammatory response, cytokines such as HSP70 and TNF-α are released and react, activating cell death receptors (2). TNF-α binds to death receptors transversing the cellular membrane activating procaspase 8 (3) with such recruitment leading to cleavage of caspase 3 (4) and cell death (5).

The current study investigated the toxicity to amorphous SiO_2_ NPs in the lungs of Golden Syrian Hamsters. The study found amorphous SiO_2_ NPs induce cell death through an extrinsic mechanism of apoptosis. The data from Golden Syrian Hamsters suggest that internalization of amorphous SiO_2_ NPs by macrophages and neutrophils may lead to the generation of TNF-α, that will bind to a transmembrane receptor, that in turn will signal the death receptor cleavage of Caspase 8 and Caspase 3 to initiate apoptosis. As this extrinsic mechanism is occurring, HSP70 is acting against the intrinsic pathway, protecting the mitochondrial membrane from permeabilizing. Resulting apoptosis may then allow for hole formation and their enlargement within the alveoli septa, seen in SEM micrographs, which allows mechanical stress to cause alveolar wall breaks, as seen by increased air space size, and quantified by mean linear intercept. Figure 9 provides a mechanistic summary.

## Data Availability

The original contributions presented in the study are included in the article/Supplementary Material, further inquiries can be directed to the corresponding author.
