# Supplementary material for: Pulmonary injury following exposure to amorphous silicon dioxide nanoparticles in Golden Syrian Hamsters
Source: Exp Biol Med (Maywood). 2026 Jan 26;251:10793. doi: 10.3389/ebm.2026.10793 (PMC12883427; doi:10.3389/ebm.2026.10793)
Supplement: Supplementary file 1 [file Supplementaryfile1.docx]

**Supplementary Material**


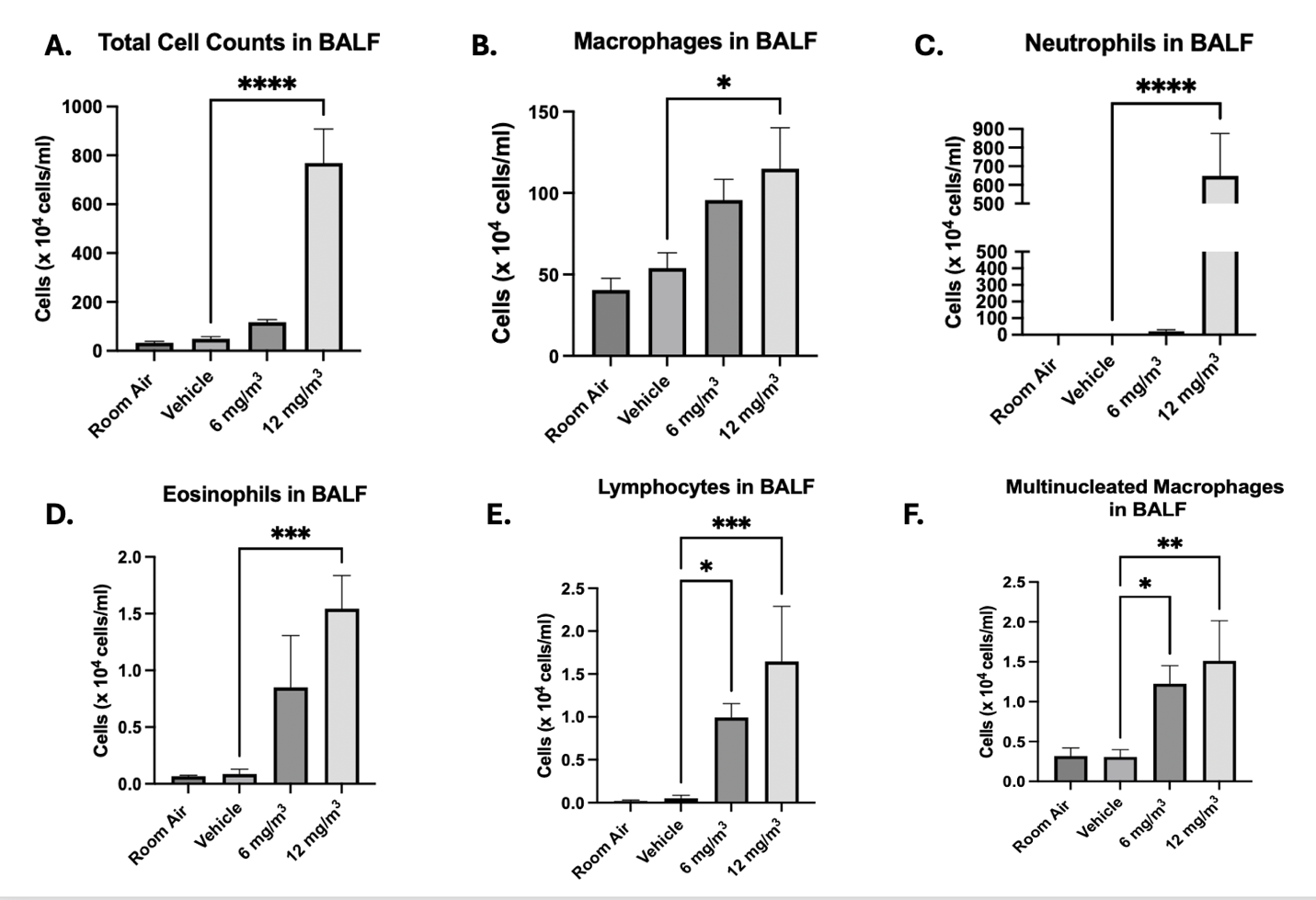


**Supplementary Figure 1.** Total Cells and leukocytes in the bronchoalveolar lavage fluid (BALF) of Golden Syrian Hamsters were counted to determine if inflammation was occurring after exposure to inhaled SiO_2_ NPs. The cells were re-suspended in 1mL of RPMI media followed by determination of total cell numbers (a). Total Cell numbers (a) were significantly increased by 16-fold in the 12 mg/m^3^ treated group. Differential cell count (b-f) slide smears were prepared from the BALF of cells from the lung exposed to 6 and 12 mg/m^3^ SiO_2_ NPs for 4h/day for 8 days. Macrophages (b) of treated groups were not changed when compared to control. Neutrophils (c), Eosinophils (d), Lymphocytes (e), and Multinucleated Macrophages (f) were significantly increased in the 12 mg/m^3^ treated group when compared to controls. N=5.


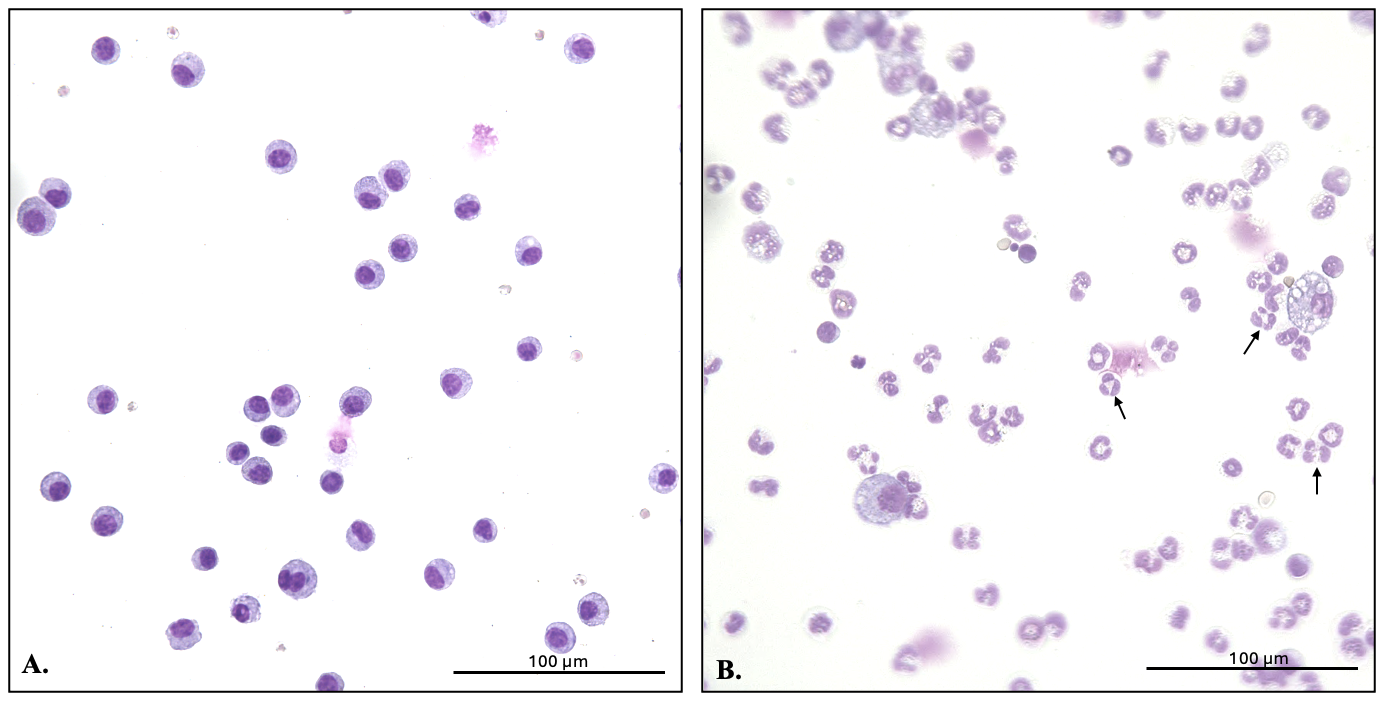


**Supplementary Figure 2.** Representative micrograph of cells from the BALF from the lungs of (a) control and (b) from 12 mg/m^3^ treated animals. (a) Note the presence of macrophages. (b) There is an increase in the number of neutrophils present indicated by arrows.

**
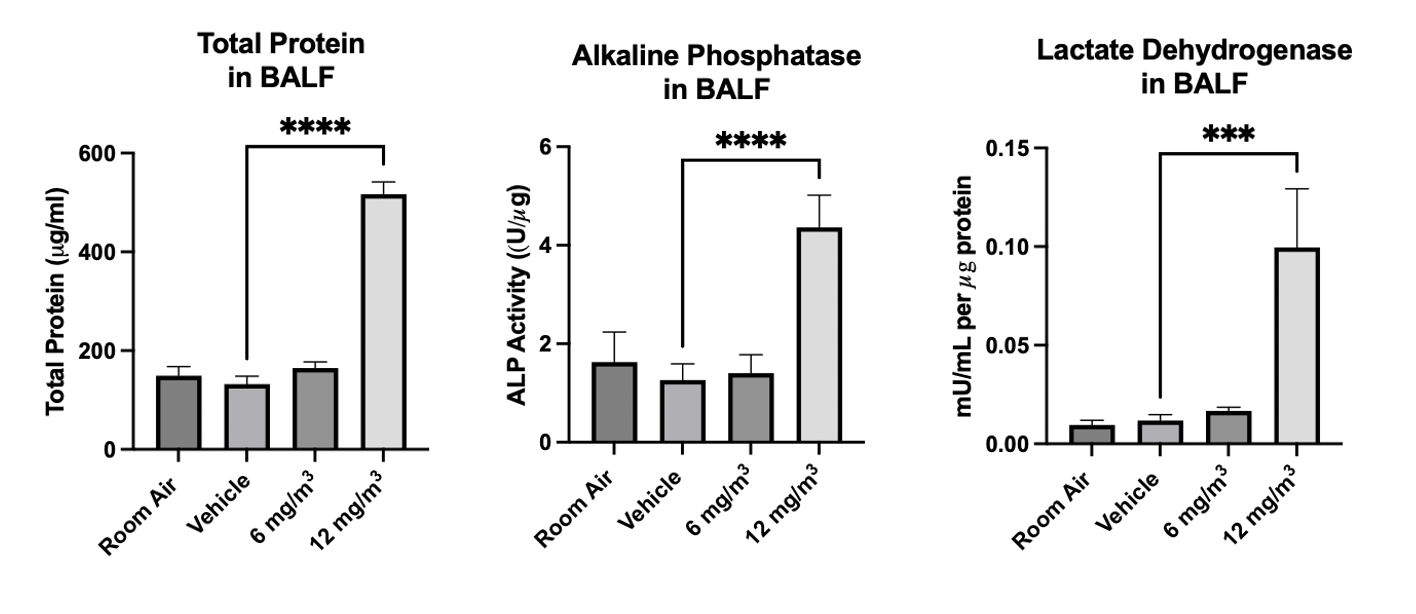
**

**Supplementary Figure 3.** Markers measured in the BALF of Golden Syrian Hamsters from lungs exposed to 6 and 12 mg/m^3^ SiO_2_ NPs for 4h/day for 8 days were Total Protein (TP) Alkaline Phosphatase (ALP), and Lactate Dehydrogenase (LDH). (a) TP, (b) ALP), and (c) LDH were all significantly increased in the 12 mg/m^3^ treated group when compared to controls. N=4.

**
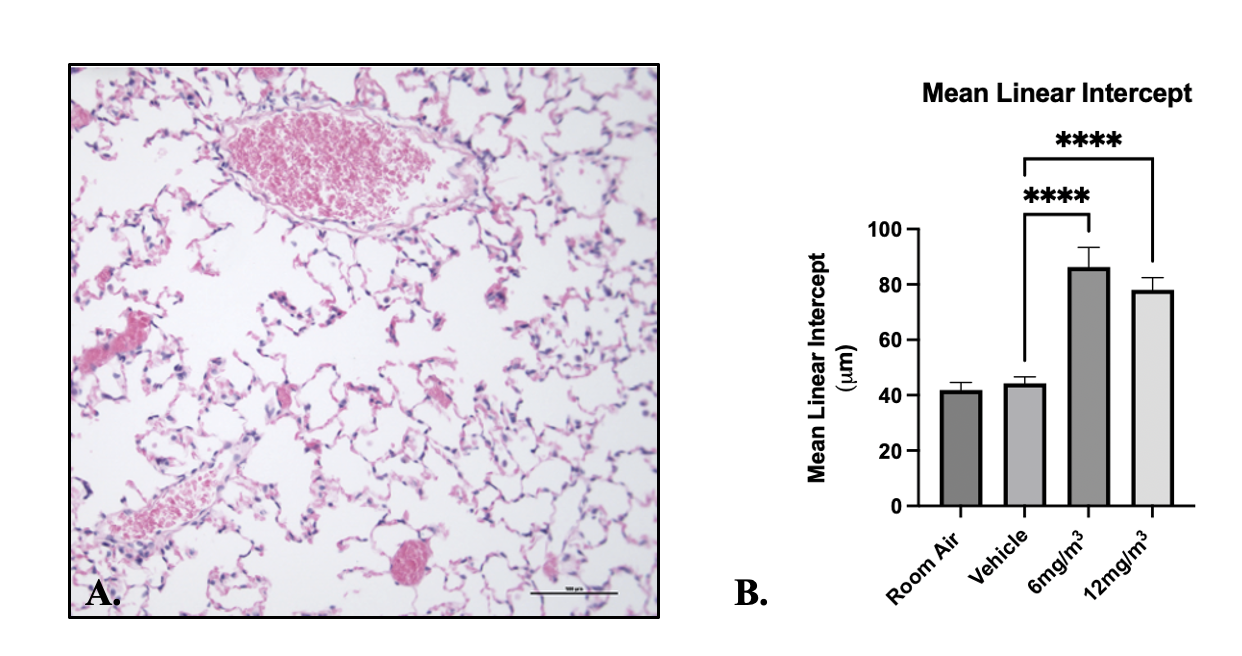
**

**Supplementary Figure 4.** The photomicrograph is of a formalin fixed, H&E-stained tissue section from the lung of (a) room air control animals. The micrograph shows a typical inflation. (B) Mean Linear Intercept was measured by the line intersection method and calculated from 20 random fields of view per animal. The Mean Linear Intercept of both 6 mg/m^3^ and 12 mg/m^3^ SiO_2_ NP treated animals was significantly increased when compared to controls.

**
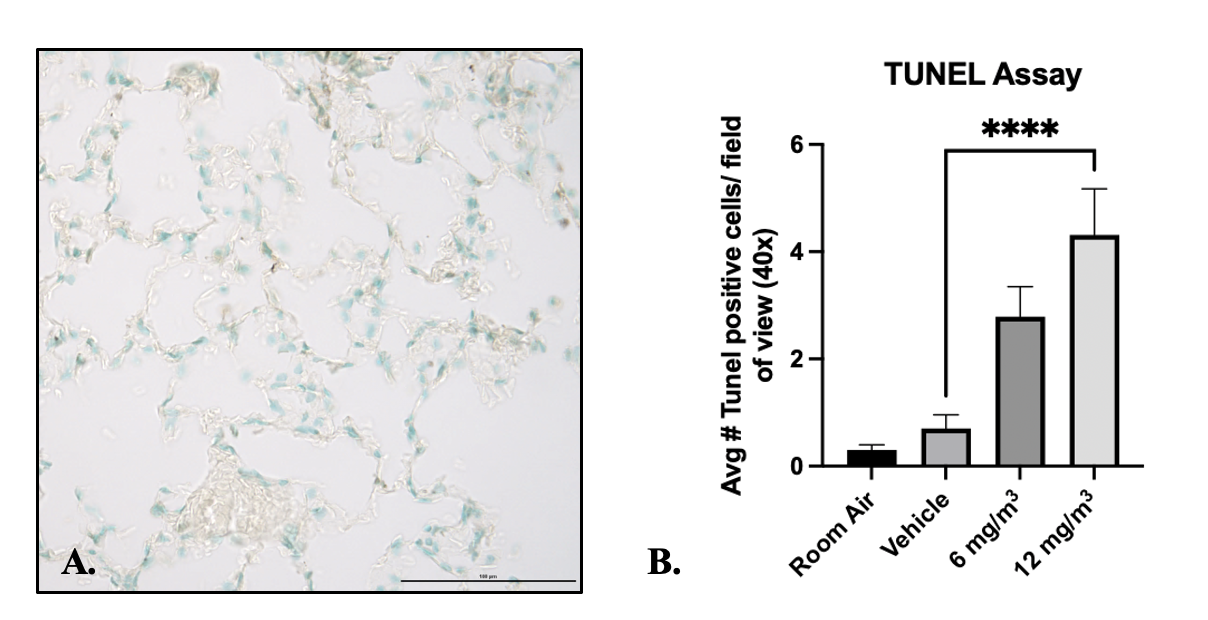
**

**Supplementary Figure 5.** The photomicrograph is of a formalin fixed tissue section from the lung of (a) room air control animal. The TUNEL Assay-stained tissue sections for TUNEL positive cells (apoptotic bodies). The micrograph shows no TUNEL positive cells within the control. (B) A histogram of the count from the TUNEL assays shows a significant increase in TUNEL positive cells in the high concentration SiO_2_ NP treated animals compared to controls. The average TUNEL positive cells was based on calculated results of 20 random fields of view.

**
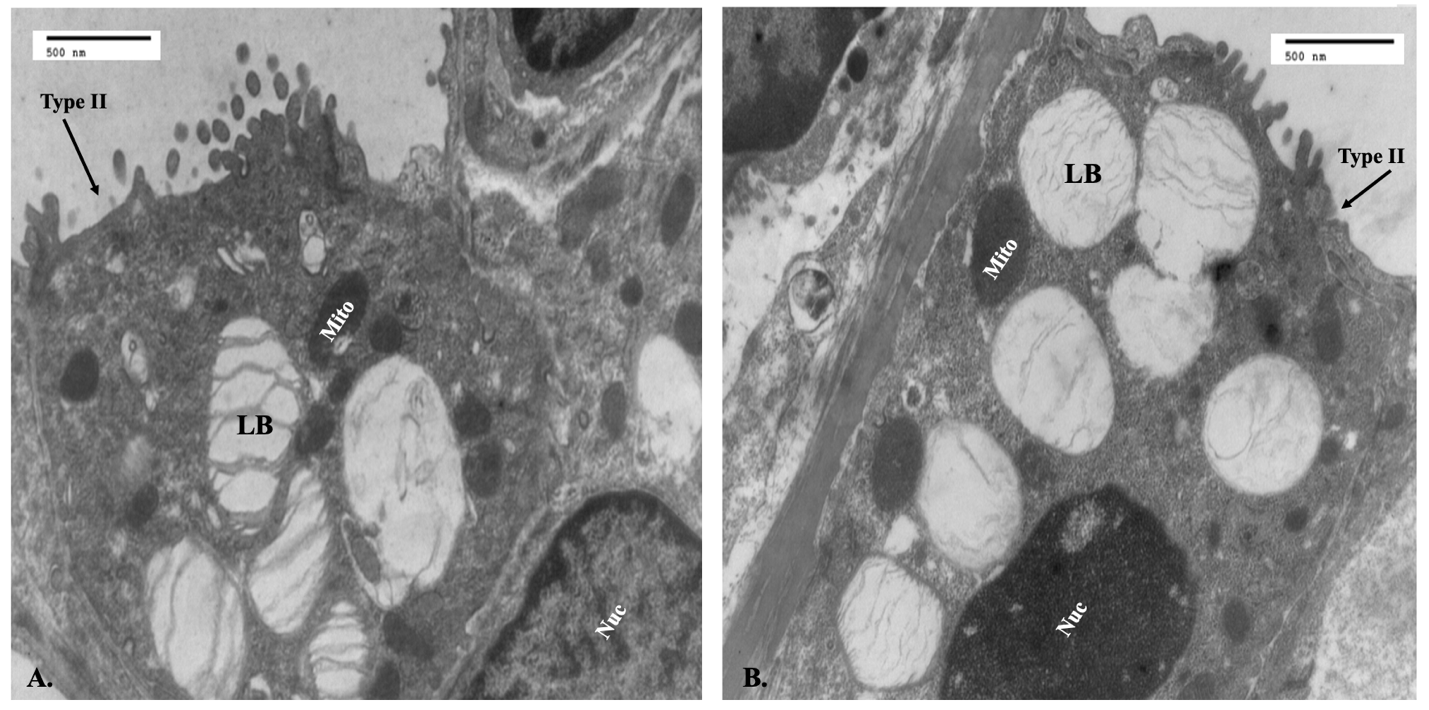
**

**Supplementary Figure 6.** Representative TEM micrographs of tissue sections from the lungs of (a) control and (b) 12 mg/m^3^ treated animals. Both (a) and (b) show a Type II cells with lamellar bodies, a nucleus, and mitochondria.

Key: Lamellar Bodie (LB), Mitochondria (Mito), Nucleus (Nuc)

**
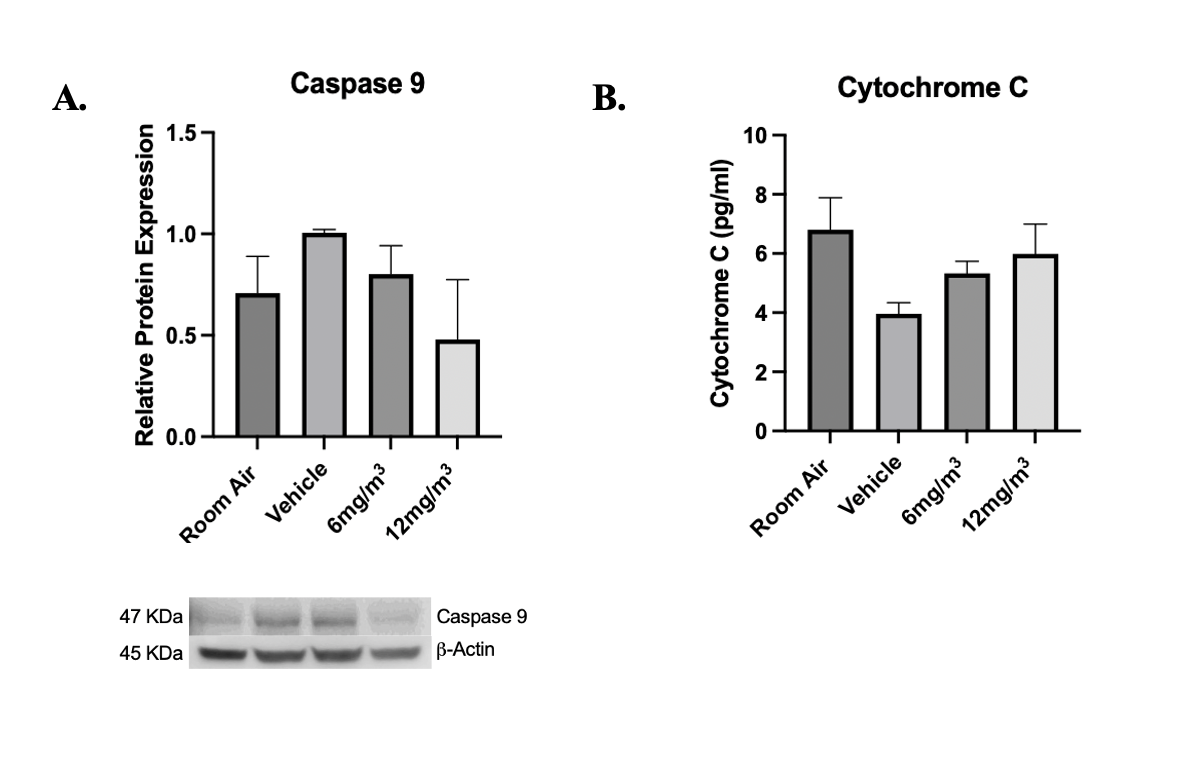
**

**Supplementary Figure 7.** Representative histogram of the Western Blot of Caspase 9 and ELISA of Cytochrome C on the tissue of controls and treated animals. (a) Caspase 9 and (b) Cytochrome C levels remained unchanged in treated animals when compared to controls. For Western Blot and ELISA n=4.

**
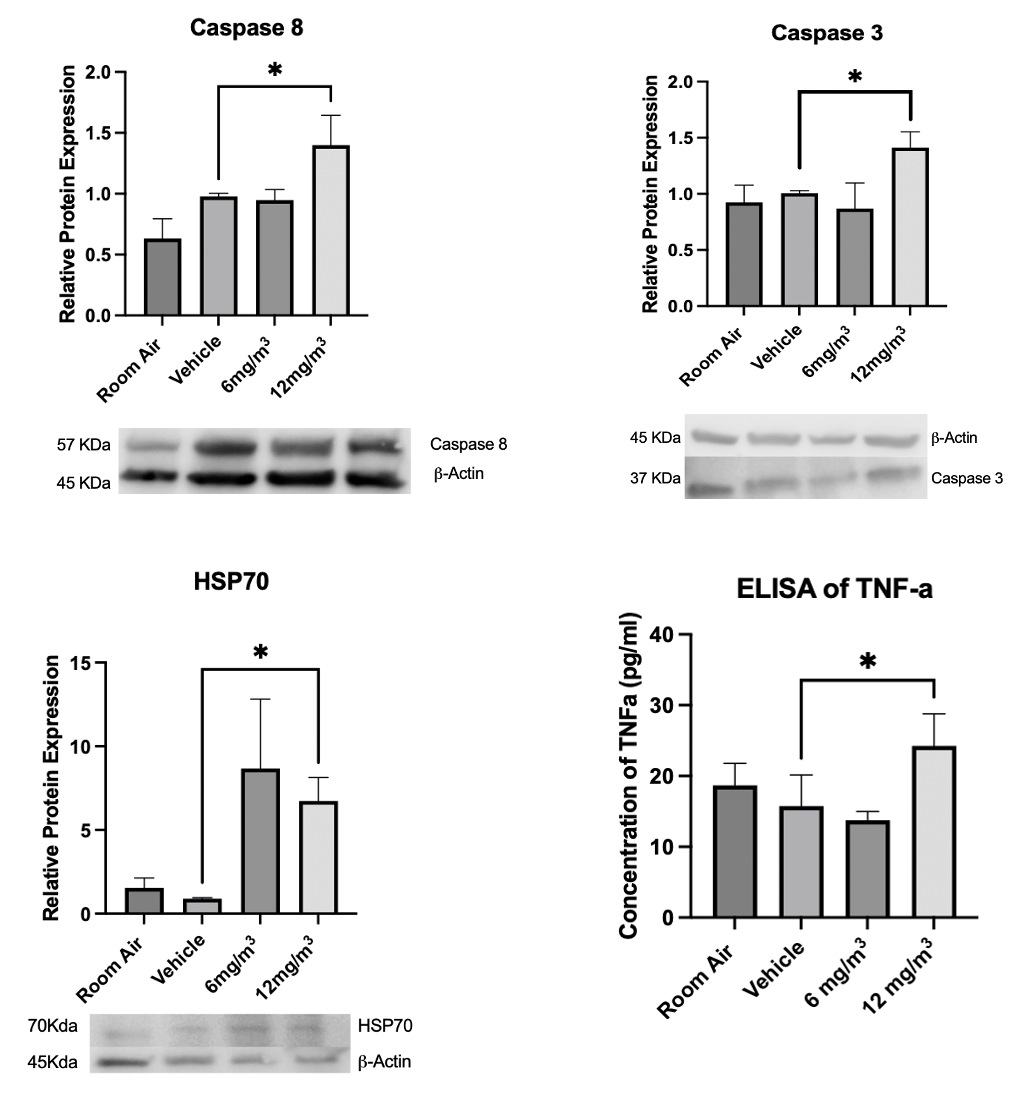
**

**Supplementary Figure 8.** To determine if apoptosis was occurring, Caspases 8, 3, HSP70 and TNF-α were measured via Western Blot or ELISA. (a) Caspase 8 and (b) Caspase 3 were significantly increased in the 12 mg/m^3^ treated group when compared to controls. (c) HSP70, a marker of cellular stress, was measured by Western Blot and was significantly increased in the high concentration group when compared to controls. (e) An ELISA of TNF-α levels was significantly increased in the high concentration treated group when compared to controls. For Western Blots and ELISAs n=4.
